# Cause of death among Ghanaian adolescents in Accra using autopsy data

**DOI:** 10.1186/1756-0500-4-353

**Published:** 2011-09-12

**Authors:** Sally-Ann Ohene, Yao Tettey, Robert Kumoji

**Affiliations:** 1 World Health Organization Country Office in Ghana, Accra, Ghana; 2 Department of Pathology, University of Ghana Medical School, Korle Bu, Accra, Ghana

## Abstract

**Background:**

There is limited data on adolescent mortality particularly from developing countries with unreliable death registration systems. This calls for the use of other sources of data to ascertain cause of adolescent mortality. The objective of this study was to describe the causes of death among Ghanaian adolescents 10 to 19 years in Accra, Ghana utilizing data from autopsies conducted in Korle Bu Teaching Hospital (KBTH).

**Findings:**

Out of the 14,034 autopsies carried out from 2001 to 2003 in KBTH, 7% were among adolescents. Of the 882 deaths among adolescents analyzed, 402 (45.6%) were females. There were 365 (41.4%) deaths from communicable disease, pregnancy related conditions and nutritional disorders. Non-communicable diseases accounted for 362 (41%) cases and the rest were attributable to injuries and external causes of morbidity and mortality. Intestinal infectious diseases and lower respiratory tract infections were the most common communicable causes of death collectively accounting for 20.5% of total deaths. Death from blood diseases was the largest (8.5%) among the non-communicable conditions followed by neoplasms (7%). Males were more susceptible to injuries than females (χ^2 ^= 13.45, p = .000). At least five out of ten specific causes of death were as a result of infections with pneumonia and typhoid being the most common. Sickle cell disease was among the top three specific causes of death. Among the females, 27 deaths (6.7%) were pregnancy related with most of them being as a result of abortion.

**Conclusions:**

The autopsy data from the Korle-Bu Teaching Hospital can serve as a useful source of information on adolescent mortality. Both communicable and non-communicable diseases accounted for most deaths highlighting the need for health care providers to avoid complacency in their management of adolescents presenting with these diseases.

## Background

Adolescence is generally perceived as being a healthy period of life [1,2]. Particularly in the developing world however the lives of adolescents are being compromised and cut short because of exposure to serious risks they are not adequately equipped to handle [3]. Patton and colleagues estimated that among young people, mortality rates were highest in Africa and Southeast Asia accounting for two-thirds of worldwide deaths among this population [4]. To ascertain the causes of these deaths in our young people, mortality data is needed. As Setel and colleagues argue, mortality data are important for planning, setting priorities and formulating and monitoring policies [5]. The irony is that whereas adolescent mortality data can be accessed from several high income countries, this is not so in developing countries due to the limitation of reliable death registration records. In WHO's Africa region, only 3 countries have vital registration coverage above 85% [6] and Ghana is not one of them. Consequently the weak death registration system limits even the use of death certificates as a major source of cause of death information. This calls for innovative ways of accessing and generating adolescent mortality data particularly from low income countries.

A potential source of data for adolescent mortality is data from hospitals. While few studies have used hospital records and autopsy data to report on causes of death, even fewer have focused on adolescent deaths [7-10]. Granja et al through a review of hospital records reported on maternal related deaths among adolescents in Mozambique and classified 75% of these deaths as avoidable [11]. While there is a limitation of bias from this data source, in the absence of adequate death registration, data from this source provides useful information on cause specific mortality and patterns. This may be useful for identifying potential areas for interventions [12]. The objective of this study was to describe the causes of death among adolescents 10 to 19 years utilizing autopsy data.

## Methods

Data for this study was derived from a retrospective analysis of autopsy records from the Korle Bu Teaching Hospital (KBTH). This hospital, the largest in Ghana with a 1400 bed capacity at the time of the study, is located in Accra the capital of Ghana and utilized by the University of Ghana Medical School. Being a referral hospital, KBTH receives cases from all over the country as well as from Accra and its surrounding suburbs with an estimated population of over four million. The mortuary in KBTH being both a hospital and a public mortuary accepts for storage and or autopsy dead bodies from both within the hospital and outside from other facilities and the communities in Accra and surrounding areas. Approximately 80% of the autopsies carried out in the KBTH mortuary are Coroner's cases. (KBTH mortuary records) Approximately 70% of autopsies requested by the Coroner in the city of Accra over the study period were conducted in KBTH and these included deaths from the hospital itself as well as from outside. (Unpublished data). These Coroner's cases by definition are deaths from unknown causes (occurring at home or outside the health facility) or deaths occurring within 24 hours of admission to a health facility, suspicious deaths and "unnatural" deaths in which the cases had no known attendance at a health facility [13]. An estimated 90% of deaths from all age groups occurring in KBTH are autopsied to address clinical problems that clinicians may have, confirm the cause of death and determine cause of death particularly since many of these deaths meet the definition of a Coroner's case. (KBTH records) Autopsies whether performed for the Coroner or for the hospital are carried out by a qualified pathologist or a trainee doctor supervised by the pathologist. The autopsy consists of a review of the history from relatives and/or from medical records (where available), an external examination, and a dissection with internal examination of all major organ systems [13]. Additional studies (histology, microbiology, toxicology) are performed in selected cases, as determined by the pathologist. The cause of death is constructed along the guidelines and structure provided in the International Classification of Diseases, ICD 10 (10^th ^Edition ICD, World Health Organization) [14]. In Coroner's cases, it is issued by the pathologist. In non-Coroner's cases, the cause of death is issued by the referring physician in consultation with the pathologist who performed the autopsy.

Results of autopsies conducted on adolescents 10 to 19 years over the 3 year period from 2001 to 2003 were compiled and entered into an Excel Sheet. Cases that had complete data on age, gender, source of referral, cause of death/underlying disease and the cause of death coded according to ICD 10 made up the study population. This data was imported into STATA Data Analysis and Statistical Software version 9 for the analysis. (Stata, Version 9, College Station, TX). The cases were grouped into two age groups; young (10 - 14 years) and older (15 - 19 years) adolescents. The data was analyzed according to sex and age group for the following three broad causes of death categories as per burden of disease groups: Group I, communicable disease mortality including infectious causes, pregnancy related deaths and nutritional disorders; Group II, non-communicable disease including neoplasms and sickle cell diseases; and Group III, injury related deaths including traffic accidents and drowning [15]. The data was also analysed to determine the top ten specific causes of death by sex and age group. Deaths for which ICD codes pointed to non-specific causes of death were allocated to appropriate groups. Clearance to conduct the study was obtained from the Ethical and Protocol Review Committee of the University of Ghana Medical School.

## Results

**Figure 1:**
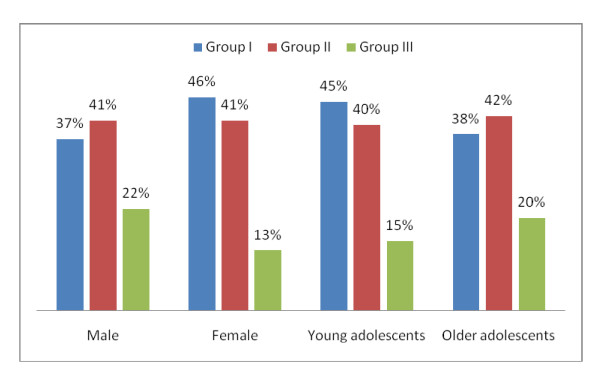
**Distribution of deaths among adolescents according to disease burden groups by sex and age-group**. Group I - communicable disease mortality including infectious causes, pregnancy related deaths and nutritional disorders; Group II - non-communicable disease including neoplasms and sickle cell diseases; Group III - injuries including road traffic accidents, other unintentional injuries and intentional injuries.

From 2001 to 2003, 927 autopsies, constituting 7% of the total of 14,034 autopsies carried out in this period in the KBTH mortuary were conducted were among adolescents 10 to 19 years. Out of the 927, complete data was available for eight hundred and eighty two. One fifth of these 882 adolescent cases autopsied originated from KBTH and the rest were from outside the hospital and different parts of Accra. Almost all the cases, 99% of the 882, were Coroner's cases. There were 402 females (45.6%) and 480 males. Three hundred and sixty four (41.3%) were young adolescents 10 to 14 years and the older adolescents were 518 (58.7%). Group I deaths, 365 in all, accounted for 41.4% of the mortality in the adolescents. These included 27 deaths from maternal causes. There were 362 deaths (41%) from Group II (non-communicable conditions). Group III deaths from injuries and other external causes of morbidity and mortality accounted for the 155 deaths (17.6%). The proportions of adolescents by gender and age group dying as a result of conditions from the 3 groups are presented in Figure 1. In bivariate analysis, it was evident that young adolescents were more affected by Group I deaths than the older adolescents (χ^2 ^= 3.97, p = .046). It also appeared that females were more affected by Group I deaths than males (χ^2 ^= 7.27, p = .007) however this difference between genders was due to the maternal deaths included in Group I. Group II causes of death affected both males and females equally and also both young and older adolescents equally. On the other hand, there was strong evidence that males were more susceptible to death from injuries than their female counterparts (χ^2 ^= 13.45, p = .000). Older adolescents were only slightly more vulnerable to death from injuries than their younger colleagues (χ^2 ^= 3.88, p = 0.049).

Table 1 shows the common broad disease conditions for Groups I and II. Intestinal infectious diseases were the most common among the communicable diseases with typhoid alone accounting for 78%. Almost all the inflammatory central nervous system cases (94%) were due to meningitis. Twenty-six out of the 27 maternal deaths occurred among the older adolescent females and 20, (about 75%) of them were a consequence of abortion. There were 8 cases of HIV, 5 of which were co-infected with tuberculosis. Two deaths were as a result of rabies.

**Table 1:** Distribution of disease categories causing death among adolescents by groups

Cause of death	Number	Group %	Overall %
**Group I**			
Intestinal Infectious Disease	93	25.5	10.5
Lower Respiratory Tract Infection	88	24.1	10.0
Inflammatory Central Nervous	50	13.7	5.7
Malaria	38	10.4	4.3
Tuberculosis	30	8.2	3.4
Maternal	27	7.4	3.1
Nutritional disorders	22	6.0	2.5
Other	17	4.7	1.9
**Total**	**365**	**100**	**41.4**
			
**Group II Non-Communicable**			
Blood Disease	75	20.7	8.5
Neoplasms	62	17.1	7.0
Cardiovascular	49	13.5	5.6
Renal and Genitourinary	49	13.5	5.6
Digestive Tract Conditions	45	12.4	5.1
Undetermined	22	6.1	2.5
Neuropsychiatric Conditions	21	5.8	2.5
Respiratory Diseases	16	4.4	1.8
Congenital Anomalies	11	3.0	1.2
Other	12	3.3	1.4
**Total**	**362**	**100**	**41.0**
			
**Group III Injuries**			
Road Traffic Accidents	50	32.3	5.7
Other Unintentional Injuries	95	61.3	10.8
Intentional Injuries (Suicide and homicide)	10	6.4	1.1
**Total**	**155**	**100**	**17.6**
			
**Total**	**882**		**100**

The leading cause of death for Group II was blood diseases of which sickle cell disease (SCD) was responsible for 95%. Among the malignant neoplasms, non Hodgkin lymphoma was the most common (28.5%) followed by malignant brain tumours (15.9%). The leading causes of death from cardiovascular, renal/genitourinary and digestive tract conditions were cardiomyopathy (20.4%), chronic glomerulonephritis (24.5%) and cirrhosis of the liver (17.8%) respectively. More than two-thirds of the Group III deaths were as a result of drowning and road traffic accidents. A more detailed description of the mortality from injuries in this study is reported elsewhere [16]. Table 2 shows the ten leading specific causes of death by age group and sex. Across these categories, the majority of deaths could be traced to infections, with pneumonia, and typhoid being the most common. Sickle cell disease was also a notable cause of death featuring among the top 3 conditions affecting older adolescents and both males and females. Drowning was first on the list for males while maternal conditions ranked fifth for females.

**Table 2:** Top ten causes of death among adolescents by age group and sex

Young Adolescents (N = 364)	N (%)	Older Adolescents (N = 518)	N (%)
Typhoid	39 (10.7)	Pneumonia	49 (9.5)
Pneumonia	38 (10.4)	Sickle cell	44 (8.5)
Malaria	28 (7.7)	Typhoid	35 (6.8)
Sickle cell	27 (7.4)	Drowning	34 (6.6)
Malignant neoplasms	23 (6.3)	Road traffic accidents	33 (6.4)
Drowning	23 (6.3)	Malignant neoplasms	32 (6.2)
Meningitis	22 (6.0)	Meningitis	25 (4.8)
Road traffic accidents	17 (4.7)	Tuberculosis	18 (3.5)
Tuberculosis	12 (3.3)	Infectious gastroenteritis	12 (2.3)
Anemia	10 (2.7)	Malaria	10 (1.9)
			
Males (N = 480)	N (%)	Females (N = 402)	N (%)

Drowning	45 (9.4)	Pneumonia	43 (10.7)
Pneumonia	44 (9.2)	Typhoid	35 (8.7)
Sickle cell	40 (8.3)	Sickle cell	31 (7.7)
Typhoid	39 (8.1)	Malignant neoplasms	28 (7.0)
Meningitis	34 (7.1)	Maternal	27 (6.7)
Malignant neoplasms	27 (5.6)	Road traffic accidents	24 (6.0)
Road traffic accidents	26 (5.4)	Malaria	19 (4.7)
Malaria	19 (4.0)	Tuberculosis	15 (3.7)
Tuberculosis	15 (3.1)	Meningitis	13 (3.2)
Infectious gastroenteritis	9 (1.9)	Drowning	12 (3.0)
		Anemia	12 (3.0)

## Discussion

This study using autopsy data from KBTH mortuary showed that both Group I and Group II conditions were dominant causes of deaths among the adolescents autopsied. There was a higher representation of males and older adolescents in the study population. At least five out of ten specific conditions leading to death were as a result of infections.

Unlike Blum's study which identified HIV as a leading cause of death among 15 to 29 year olds in Africa followed by infections, infections were an important cause of death among the adolescents in our study but not HIV [17]. Besides the difference in the age range in our study population, Ghana has a relatively low HIV prevalence compared to many of the southern African countries which may help explain the difference between our findings and those in Blum's report [18]. Intestinal infectious diseases particularly, typhoid and lower respiratory tract infections, the most common communicable diseases resulting in death in our study population are among the identified leading causes of death in the general population in Ghana, the African region and globally [4,6,16,19,20]. The paucity of adolescent and country specific aggregated data in these reports however make it difficult to compare whether the ranking of these conditions are similar. Ghosh et al in an age-wise analysis of the distribution of typhoid fever found adolescents 10 to 14 years to be more vulnerable [21]. They speculated that contributing factors included less restrictive attention at this age and an increased consumption of unhygienic food and water which predisposed them to typhoid infection.

Pneumonia, long recognized as a major childhood killer, is also reported to have a significant impact on adolescent health [22]. Our study findings corroborated Patton's by identifying lower respiratory tract infections (RTI) as a common cause of death in our adolescents [4]. With death rates from respiratory infections highest in sub-Saharan Africa, prompt management of RTI among adolescents is also imperative to reduce their risk of succumbing [23].

Among the non-communicable diseases affecting the adolescents in our study, sickle cell disease (SCD) featured prominently. Even though the literature shows that mortality from SCD usually occurs in adulthood, adolescents are also vulnerable to death from this disease [24,25]. Loureiro and colleague in their evaluation of deaths from SCD found in one locality in Brazil that, mortality was concentrated among children less than 4 years [24]. This was followed by a decline and subsequently a rise beginning at age 15 years. From an autopsy study of SCD patients, Manci and colleagues found infections to be the most common cause of deaths in all age groups [25]. The susceptibility of adolescents to infections noted in this study thus highlights the need to also manage adolescents with SCD with vigilance.

The most common cause of death from cancer in our population was that originating from hematopoietic organs. This is similar to findings from a cancer mortality study carried out in Ghana, in which among young people 15 to 24 years, malignancies of the hematopoietic system dominated the picture for both males and females [26]. Pulte and colleagues point out that though there has been improvement in survival rates for adolescent with hematological cancers, children and with some particular hematological cancers, even adults have a higher survival than adolescents [27]. They suggested that the worse outcome for those 15 to 24 years may be linked to social issues affecting this age group as well as other factors such as lower compliance rates. Brain cancer which was the next most common cause of cancer deaths in our study was also second and fourth in position for females and males respectively in Wiredu's study [26].

In our study population, injuries were responsible for less than a fifth of the deaths in our study compared to a range of 30 to 80% in high income countries [4,28,29]. The speculation may be that since injury deaths are less likely to end up in hospital, this relatively low figure in our study could be attributed to the bias of using data from a hospital with the inherent problems of under-reporting injury deaths. While this may be so, it important to note that in Blum's report, injuries caused relatively fewer deaths in African youths compared to their counterparts in other regions of the world [17]. Besides in Ghana, since all injury deaths are Coroner's cases they are more likely to be autopsied irrespective of where the injury death occurred. As observed in our study, practically all the autopsies conducted in our study, including all the injury deaths, were Coroner's cases. Regardless of the relatively lower proportion of injury deaths, there is still cause for alarm because these deaths, most of which were from drowning and road traffic accidents can be prevented if appropriate road safety measures, education on safety around water bodies and an integrated approach to injury prevention regulations are instituted [30].

Several of the top 10 specific causes of death in our study were similar to those reported for adolescents worldwide though to varying degrees [4]. The presence of several infectious causes in this list calls for a heightened awareness of the vulnerability of adolescents to infections. Given that adolescents are generally perceived to be healthy there is a need to avoid complacency in managing infections that may be contracted. The proportion of females who died from maternal causes is comparable to what has been reported [4]. In Sub-Saharan Africa abortion is recognized as being responsible for a significant proportion of these maternal deaths confirming our findings [31]. This re-iterates the need to provide comprehensive sexual and reproductive health information and services to help reduce these deaths [32].

The limitation of this study lies in the use of autopsy data from the mortuary of the main hospital in Accra to describe cause of deaths among adolescents. The issue of selection bias poses a challenge when using hospital data especially in the absence of a robust death registration system to confirm findings. Only a fifth of the autopsied cases in this study were from the hospital while the rest were from different parts of Accra and also almost all the cases were Coroner's cases suggesting that practically all deaths in this age range 10 to 19 years are autopsied. Nevertheless hospital data is generally perceived to under-report the real situation so caution must be exercised when interpreting the study results as the extent of representativeness of the actual picture of adolescent mortality cannot be verified. Consequently, the findings preclude generalization to all Ghanaian adolescents even though the study results are similar to what has been reported elsewhere. Further research comparing similar data sources from other parts of the country and looking at trends over the years will widen the scope of available data to provide needed adolescent mortality information.

In conclusion, in the absence of a functional vital death registration system and the limited availability of death certificates as a reliable source of information on deaths among 10 to 19 year olds, this study has shed some light on the causes of mortality in adolescents that are referred for autopsy. Several of these causes also affect other age groups including children under five. Thus in this era of competing demands for limited resources there might be the tendency to place adolescent mortality low on the priority list of health policy makers and planners. It is however important to note that not only do adolescents have a right to attain the highest standard of health; they also are the next generation of parents, workforce and leaders [32]. Investing in their health and reducing mortality from causes that are preventable or treatable will promote a healthy generation of adults who will in turn safeguard the health of the next generation.

## List of abbreviations

HIV: Human Immunodeficiency Virus; ICD: International Classification of Diseases; KBTH: Korle Bu Teaching Hospital; RTA: Road traffic accidents; RTI: Respiratory Tract Infections; SCD: Sickle Cell Disease; WHO: World Health Organization.

## Competing interests

The authors declare that they have no competing interests.

## Authors' contributions

YT and SAO conceptualized the study. YT and RK compiled and entered the data and SAO drafted the manuscript including the analysis. All authors read, edited and approved the final manuscript.
